# New *Pseudomonas* spp. Are Pathogenic to Citrus

**DOI:** 10.1371/journal.pone.0148796

**Published:** 2016-02-26

**Authors:** Farid Beiki, Antonio Busquets, Margarita Gomila, Heshmat Rahimian, Jorge Lalucat, Elena García-Valdés

**Affiliations:** 1 Iranian Research Institute of Plant Protection, Agricultural Research Education and Extension Organization (AREEO), Tehran, Iran; 2 Department of Biology-Microbiology, Universitat de les Illes Balears, Palma de Mallorca, Crtra. Valldemossa km 7.5, 07122 Palma de Mallorca, Spain; 3 Agricultural Sciences and Natural Resources University Sari, Māzandarān, Iran, Department of Plant Protection, Sari Agricultural Sciences and Natural Resources University, P.O. BOX 578, Mazandaran, SaSari, Iran; 4 Institut Mediterrani d’Estudis Avançats (IMEDEA, CSIC-UIB), Crtra. Valldemossa km 7.5, 07122 Palma de Mallorca, Spain Palma de Mallorca, Spain; Virginia Tech, UNITED STATES

## Abstract

Five putative novel *Pseudomonas* species shown to be pathogenic to citrus have been characterized in a screening of 126 *Pseudomonas* strains isolated from diseased citrus leaves and stems in northern Iran. The 126 strains were studied using a polyphasic approach that included phenotypic characterizations and phylogenetic multilocus sequence analysis. The pathogenicity of these strains against 3 cultivars of citrus is demonstrated in greenhouse and field studies. The strains were initially grouped phenotypically and by their partial *rpoD* gene sequences into 11 coherent groups in the *Pseudomonas fluorescens* phylogenetic lineage. Fifty-three strains that are representatives of the 11 groups were selected and analyzed by partial sequencing of their 16S rRNA and *gyrB* genes. The individual and concatenated partial sequences of the three genes were used to construct the corresponding phylogenetic trees. The majority of the strains were identified at the species level: *P*. *lurida* (5 strains), *P*. *monteilii* (2 strains), *P*. *moraviensis* (1 strain), *P*. *orientalis* (16 strains), *P*. *simiae* (7 strains), *P*. *syringae* (46 strains, distributed phylogenetically in at least 5 pathovars), and *P*. *viridiflava* (2 strains). This is the first report of pathogenicity on citrus of *P*. *orientalis*, *P*. *simiae*, *P*. *lurida*, *P*. *moraviensis* and *P*. *monteilii* strains. The remaining 47 strains that could not be identified at the species level are considered representatives of at least 5 putative novel *Pseudomonas* species that are not yet described.

## Introduction

The plant pathogenic *Pseudomonas* species that belong to the *Pseudomonas syringae* species complex include *P*. *cannabina*, *P*. *avellanae*, *P*. *amygdali*, *P*. *ficuserectae*, *P*. *savastanoi*, *P*. *tremae*, *P*. *meliae*, *P*. *caricapapayae* and *P*. *syrin*gae [(ISPP Taxonomy of Plant Pathogenic Bacteria Committee; http://www.isppweb.org/names_bacterial.asp); [1]]. *P*. *syringae* is the first species in the Top 10 plant pathogenic bacteria [2]. Traditionally, these pathogens have been differentiated from other *Pseudomonas* species according to their colony morphology, ability to induce a hypersensitivity response in non-host plants, and the presence or absence of pectinase and arginine dihydrolase [3]. Some authors have also included the pectinolytic species *P*. *viridiflava* and the oxidase-positive species *P*. *cichorii* within this group [4]. Both species groups are monophyletic in the *rpoD* gene’s phylogenetic tree and in multilocus sequence analysis (MLSA) and are considered members of the *P*. *syringae* phylogenetic group as defined by Mulet and collaborators [5].

*P*. *syringae* and *P*. *viridiflava*, which cause citrus blast and black pit disease, are pathogenic species to citrus plants. Blast is a disease of the leaves and twigs, and black pit is a disease of the fruit. *P*. *syringae* and *P*. *viridiflava* are widespread on citrus foliage, although their presence does not always lead to disease development. In general, the occurrence of disease symptoms is influenced by several factors, such as temperature, humidity, oxygen depletion, varietal susceptibility and the virulence of the bacterial strains. Under favorable conditions, the disease develops very quickly and could cause substantial economic losses [2]. These diseases are widespread in Iran under cool and wet conditions. Citrus blast is one of the most important citrus diseases in the northern citrus growing provinces of Iran, which represents 60% of the total citrus growing area in Iran (approximately 100,000 ha). Damage is mainly caused by the prevailing climatic conditions in this area of the Caspian Sea belt [6, 7, 8]. The disease has caused considerable damage to citrus orchards in recent years in this area, although it has not caused much damage in the other orchards in Iran. No exact crop loss data are available. Farmers use bactericidal compounds to control the disease (e.g., copper oxychloride); however, this practice could cause serious damage to the environment and human health and also promotes the selection of pathogenic strains with increased tolerance to copper [9, 10]. Symptoms of the disease, initially water-soaked lesions turning to brown to black necrotic areas, most commonly begin on young leaves and twigs. Leaf lesions extend through the petiole to the stem and expand in both directions. Expansion of the lesions often leads to girdling of the affected branch and withering of the portions distal to the lesion [11].

Differentiation of species in the *P*. *syringae* species complex by phenotypic characteristics, 16S rDNA phylogenetic analysis and cell wall fatty acid composition lacked the required resolution for reliable differentiation of the taxa [12, 13, 14]. Recently, the convenience of sequence-based analysis for rapid and precise identification of plant pathogenic *Pseudomonas* has been proposed by several authors [15, 16, 17]. Partial sequences of the *rpoD* gene have been proposed for the differentiation of *Pseudomonas* species, with a species cut-off at 95% sequence similarity [18, 5].

More than 1,000 strains were isolated in an initial screening from samples of citrus leaves and twigs with blast disease symptoms collected from different regions of Iran (Gilan, Mazandaran and Golestan provinces). One hundred and forty strains were positive in the pathogenicity tests conducted, and the strains that were phenotypically related to *Pseudomonas* strains (126) were included in the present study. The collection of 126 *Pseudomonas* strains has been identified and characterized taxonomically by extensive numerical analyses of phenotypic features and by a multilocus sequencing approach, which includes analyses of partial sequences of the 16S rRNA, *gyrB* and *rpoD* genes. Strains of *Pseudomonas* species previously not considered as pathogenic to plants (*P*. *simiae*, *P*. *orientalis*, *P*. *moraviensis* and *P*. *monteilii*) and at least five putative novel species were isolated and characterized taxonomically by means of a polyphasic approach; the pathogenic properties of these strains to citrus were demonstrated in the present study.

## Materials and Methods

### Sampling sites

Citrus orchards in Northern Iran cover an area located in the Caspian Sea belt (in Golestan, Mazandaran and Gilan provinces); this area extends in the north over 400 km from east to west and lies between the shore of the Caspian Sea and the first slopes of the Alborz mountain range. These provinces are geographically divided into two parts: the coastal plains and the mountainous areas. The Alborz Mountain Range surrounds the coastal strip and plains of the Caspian Sea like a huge barrier. The Gilan province has a humid subtropical climate with the heaviest rainfall in Iran (1,900 mm rainfall, humidity reaches up to 90% in summer with temperatures of over 26°C); the Mandazaran province has a moderate subtropical climate (1,200 mm average rainfall and 25°C in summer and 8°C in winter); the Golestan province has a moderate and humid climate (average annual temperature 18.2°C and annual rainfall 556 mm).

### Bacterial strains

One hundred twenty-six *Pseudomonas* strains were isolated from diseased citrus leaves and stems. No specific permission from any organization in Iran was required for the isolation of these bacterial strains because the research was for a PhD thesis. The field studies did not involve endangered or protected species. A segment of the leaf or twig lesion was surface sterilized in 0.5% sodium hypochlorite for two minutes and washed three times with sterile distilled water (SDW). The segment was cut into small pieces in drops of SDW, left for 10–20 min in a sterile laminar air flow cabinet and drops of the suspension were streaked on plates of nutrient agar (NA) medium (Merck, Germany). The streaked plates were incubated at 25°C. Single colonies were cultured on NA containing 1% sucrose (NAS), and after two days of growth, the plates were maintained at 4–6°C for short-term storage and routine use. Suspensions in SDW were stored at room temperature. For long storage periods, the cells were maintained in 25% glycerol at -70°C. The origins and geographical locations of the strains are indicated in S1 Table.

### Physiological and biochemical tests

Biochemical characteristics and carbon source utilization were tested using API 20NE strips (bioMérieux, Marcy l’Etoile, France) and the Biolog GN2 microplates MicroLog System (Biolog Inc. Hayward, California, U.S.A.) according to the manufacturer’s instructions. Fluorescent pigment production was tested on King B medium (Merck, Germany) [19]. Numerical analyses of the phenotypic tests were performed using the computer program MVSP (Multi-Variate Statistical Package, version 3.22, Kovach Computing Services, Anglesey, UK). A similarity matrix was generated using the Simple Matching Coefficient, and a dendrogram was constructed using the unweighted pair group method with arithmetic averages (UPGMA).

### Pathogenicity tests on citrus plants

Three different citrus cultivars were used in the pathogenicity tests: Alemow (*Citrus macrophylla*, the most sensitive cultivar in an extensive natural infection in 1998 in northern Iran) and the two most abundant cultivars in the region, Washington navel (*Citrus sinensis*) and Sour Orange (*Citrus aurantium*). Other plants were not tested for susceptibility. Five month old seedlings were maintained in a greenhouse (15°C and high relative humidity) and used for the pathogenicity tests. Forty-one strains were also tested under field conditions on new twigs or leaves at temperatures between 8–20°C and prolonged wetting by rain or fog. The strains were grown on NA at 25°C for 24 h, and the cells were suspended in sterile distilled water. Bacterial suspensions were adjusted to an optical density of 0.2 at 620 nm, corresponding to approximately 1 × 10^8^ CFU/ml, as determined by dilution plating. One hundred microliters of bacterial suspension was injected into the intercellular space of orange leaves with a 0.5 mm needle and syringe [20]. All pathogenicity tests were conducted at least twice. Control plants were treated with sterile distilled water. The inoculated plants were examined after two weeks. Disease severity was measured in mm^2^ of the necrotic lesion. Re-isolation from the representative isolates on plates of NA was performed 15–21 days after inoculation. The results were statistically analyzed with Sigma Plot version 11 and plotted as boxes and whiskers. This plot provided summary statistics for five values: the minimum, the maximum, the median, the 25th percentile, and the 75th percentile. LOPAT tests were performed as previously described: levan production [20], oxidase test [21], potato rot symptoms [22], arginine dihydrolase test and tobacco hypersensitivity reaction [22]. The reaction of the isolates in the LOPAT tests and the utilization of selected carbon compounds were determined to verify the identity of the isolates recovered compared with the inoculum, proving Koch postulates.

### DNA extraction, PCR amplification and DNA sequencing conditions

For DNA extraction, a bacterial suspension was prepared in 500 μl of 0.2 mM EDTA, 30 μl of 1 M NaOH was added, and the sample was boiled for 5 min. After 2 min centrifugation at 16.000 x g, the supernatant was recovered. PCR amplification was performed with a DNA thermocycler (Eppendorf). Each reaction mixture contained 5 μl of PCR buffer (EG Healthcare) and 5 μl of each of the four deoxynucleoside triphosphates (Roche) at a final concentration of 200 μM each. A total of 2.5 μl of each primer was used at a concentration of 10 μM, with 5 U of Taq DNA polymerase (EG Healthcare), in a total volume of 50 μl. The cycling conditions for the *rpoD* (PsEG30F/PsEG790R) [18], 16S rRNA (16F27/16R1492) [23] and *gyrB* (BAUP2/APrU) genes [24] included a denaturation period at 94°C for 5 min, followed by 30 cycles of amplification (denaturation at 94°C for 1 min, primer annealing at 55°C (48°C for *rpoD*) for 1 min, and primer extension at 72°C for 1.5 min). A final elongation step was carried out at 72°C for 10 min. The amplified products were purified with MultiScreen HTS PCR 96-well filter plates (Millipore) according to the manufacturer’s instructions. Sequencing reactions were performed using ABI Prism BigDye Terminator version 3.1, and the sequences were read with an automatic sequence analyzer (3130 genetic analyzer; Applied Biosystems).

### Phylogenetic analysis

The sequence analysis procedures were performed as previously described by Mulet and collaborators [18]. Individual trees were generated for the 16S rRNA, *gyrB*, and *rpoD* partial gene alignments. An analysis of three concatenated genes (16S rRNA, *gyrB*, *rpoD*; 2,818 nucleotides in total) was performed as described by Mulet et al. [25]. A concatenated gene tree was constructed with individual alignments in the following order: 16S rRNA, *gyrB* and *rpoD*. The length and nucleotide positions are in reference to *P*. *aeruginosa* type strain DSM 50071^T^: the 16S rRNA gene (X06684) nucleotide positions from 98 to 1372; the *gyrB* gene (AB039386) nucleotide positions from 326 to 1122; and the *rpoD* gene (AB039607) nucleotide positions from 94 to 743.

### Nucleotide sequence accession numbers

The nucleotide sequences determined in this study have been deposited in the EMBL database under the following accession numbers: the 16S rRNA gene from HG805683 to HG805808; the *gyrB* gene from HG805628 to HG805682; and the *rpoD* gene from HG805502 to HG805627. All sequence accession numbers used in this article are shown in S1 Table.

## Results

### Strain isolation and pathogenicity tests

**Fig 1:**
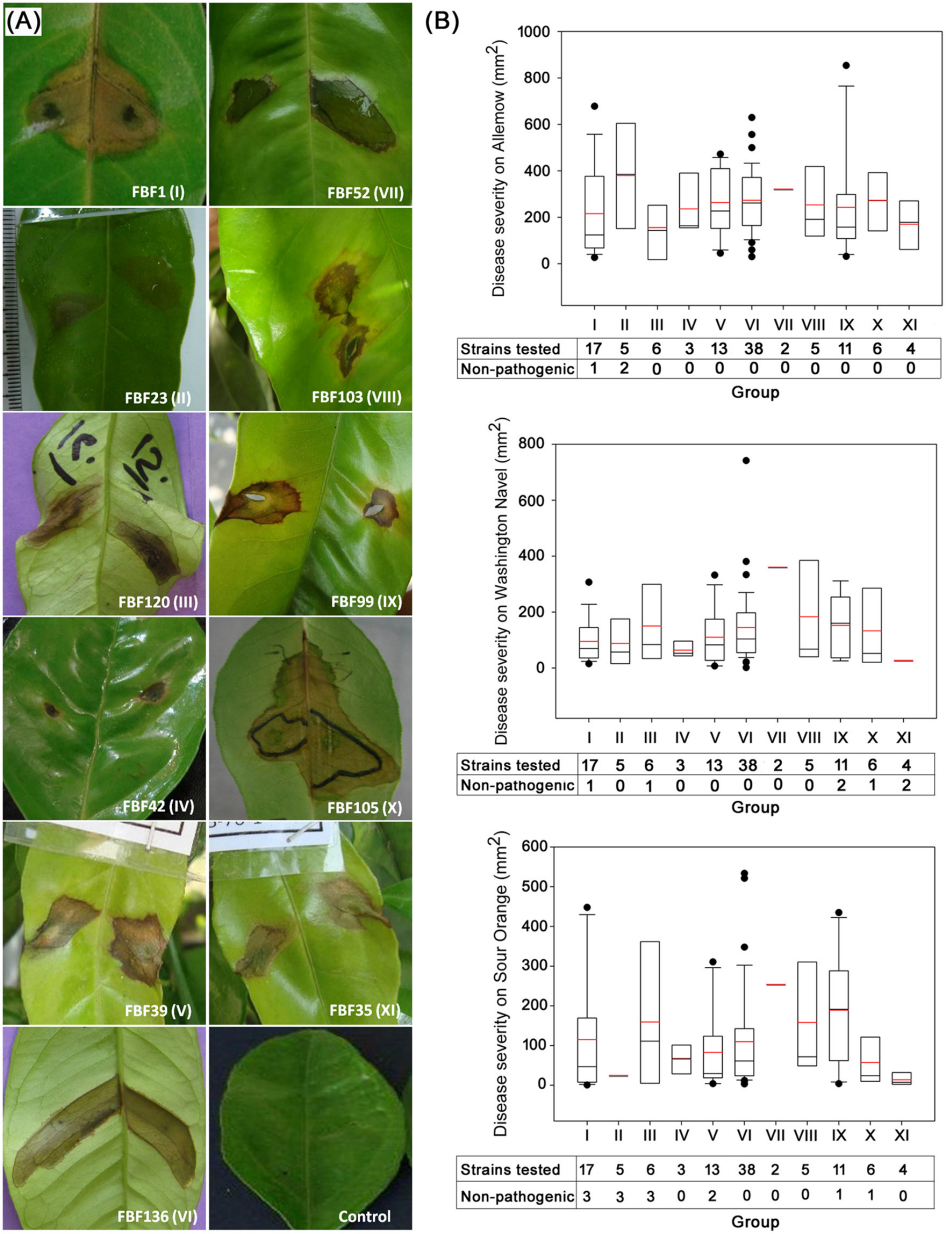
Pathogenicity tests and disease severity comparison for all *Pseudomonas* strains analyzed. **A**. Pathogenicity test in Sour Orange citrus leaves for each group of strains. **B**. Disease severity comparison of *Pseudomonas* strains of each group tested in three plant genotypes, measured by the lesion area expressed in mm^2^ two weeks after inoculation. The number of strains and the number of non-pathogenic strains for each group tested are indicated below each graph. The black horizontal line represents the median and the red horizontal line represents the mean values (if only 2 values are considered, mean and median values are identical); the boundary of the box closest to zero indicates the 25^th^ percentile and the boundary farthest from zero indicates 75^th^ percentile. Whiskers (error bars) above and below the box indicate the 90^th^ and 10^th^ percentiles. The black dots indicate outlier values.

More than 1,000 strains were isolated in an initial screening from samples of citrus leaves and twigs with blast disease symptoms collected from different regions of Iran (Gilan, Mazandaran and Golestan provinces) in 2009–2010. One hundred and forty strains were positive in the pathogenicity tests conducted, and the strains phenotypically related to *Pseudomonas* strains (126) were studied further (Table 1, S1, S2 and S3 Tables). The 126 strains were able to induce lesions in citrus leaves under greenhouse conditions and were selected for characterization. Forty-one of the isolates were tested and produced disease symptoms under natural field conditions with a monthly average temperature of 21°C. The symptoms were less severe under field conditions. There was a considerable difference in virulence among strains of the same group as depicted by the diameter of the lesion and the rate of expansion of necrosis. Disease severities in greenhouse experiments of the 126 strains distributed in 11 phenotypic and phylogenetic groups (I to XI, see below) are depicted in Fig 1 and S3 Table. The highest median severity, measured in mm^2^ of the lesion, was found among strains in group VII of the *P*. *syringae* phylogenetic group. In the majority of cases (84%), the Alemow cultivar was less resistant than the other two cultivars tested (S3 Table). As examples, results of the tests of 11 strains, one of each group in the greenhouse experiments are shown in Fig 1.

**Table 1:** Strains used in this study and their assignation to phenotypic clusters and phylogenetic groups in the *rpoD* gene tree and in the MLSA analysis.

Strain	Phenotypic cluster and *rpoD* group	*rpoD* gene closest type strain	Similarity (%)	Phylogenetic group (3 genes)	Closest type strain (3 genes)	Similarity (%)
**FBF1, FBF9, FBF10, FBF11, FBF17, FBF34, FBF43, FBF48, FBF51, FBF64, FBF66, FBF80, FBF81, FBF84, FBF86, FBF95**	I	*P*. *orientalis* DSM 17489^T^	97.8	I	*P*. *orientalis* DSM 17489^T^	97
**FBF7, FBF8, FBF23, FBF41, FBF56, FBF140, FBF141, FBF142, FBF143, FBF144**	II	*P*. *synxantha* LMG 2335^T^/ *P*. *veronii* LMG 17761^T^/ *P*. *libanensis* CIP105460^T^	92–93.7	II	*P*. *synxantha* LMG 2335^T^	96
**FBF110, FBF112, FBF120, FBF121, FBF126, FBF128, FBF130**	III	*P*. *simiae* OLI^T^	99.7–99.8	III	*P*. *simiae* OLI^T^	99.96
**FBF5, FBF20, FBF31, FBF42**	IV	*P*. *lurida* P513/18^T^	99.5	IV	*P*. *lurida* P513/18^T^	99.6–99.7
**FBF25**	I and IV^a^	*P*. *lurida* P513/18^T^	99.5	IV	*P*. *lurida* P513/18^T^	99.7
**FBF21, FBF22, FBF28, FBF30, FBF38, FBF39, FBF53, FBF54, FBF55, FBF59, FBF73, FBF75, FBF93**	V	*P*. *rhodesiae* LMG 17764^T^	91.7	V	*P*. *marginalis* ATCC 10844^T^/ *P*. *grimontii* CIP 106645^T^	94.8–94.9
**FBF12, FBF32, FBF61, FBF62, FBF69, FBF104, FBF119**	VI-A	*P*. *syringae* ATCC 19310^T^	97.1–97.8	nd	nd	
**FBF16, FBF33, FBF36, FBF40, FBF60, FBF68, FBF77, FBF78, FBF79, FBF83, FBF97, FBF106, FBF113, FBF115, FBF116, FBF139**	VI-B	*P*. *syringae* ATCC 19310^T^	96.8–98.2	VI	*P*. *tremae* LMG 22121^T^	97.1–97.3
**FBF124, FBF136**	VI-B and VI-C^a^	*P*. *syringae* ATCC 19310^T^	97.8	VI	*P*. *tremae* LMG 22121^T^	97.3
**FBF13, FBF71, FBF72, FBF74, FBF98, FBF107, FBF108, FBF118, FBF134**	VI-C	*P*. *syringae* ATCC 19310^T^	97.1–97.5	VI	*P*. *tremae* LMG 22121^T^ */ P*. *syringae* ATCC 19310^T^	97.1
**FBF27, FBF46, FBF47, FBF63, FBF111, FBF138**	VI-D	*P*. *syringae* ATCC 19310^T^	99.7–99.8	VI	*P*. *syringae* ATCC 19310^T^	99.3–99.4
**FBF109, FBF125, FBF135**	VI-E	*P*. *syringae* ATCC 19310^T^	97.2–98.0	VI	*P*. *syringae* ATCC 19310^T^	98.6
**FBF49, FBF82, FBF91**	VI-F and VI-B^a^	*P*. *syringae* ATCC 19310T	98.2–99.5	nd	nd	
**FBF52, FBF100, FBF117**	VII	*P*. *viridiflava* ATCC 13223^T^	91.8–97.8	VII	*P*. *viridiflava* ATCC 13223^T^	95.6–99.3
**FBF24, FBF58, FBF102, FBF103, FBF122**	VIII	*P*. *syringae* ATCC 19310^T^	85–85.4	VIII	*P*. *meliae* CCUG 51503^T^ / *P*. *tremae* LMG 22121^T^	91.6–91.7
**FBF2, FBF65, FBF67, FBF85, FBF87, FBF88, FBF89, FBF90, FBF92, FBF96, FBF99**	IX	*P*. *moraviensis* DSM 16007^T^	93.6–97.2	IX	*P*. *moraviensis* DSM 16007^T^/ *P*. *koreensis* LMG 21318^T^	96.2–97.2
**FBF15, FBF50, FBF57, FBF101, FBF105, FBF114**	X	*P*. *monteilii* ATCC 700476^T^	94–99	X	*P*. *monteilii* ATCC 700476^T^	95.8–98.9
**FBF18, FBF19, FBF35, FBF44**	XI	*P*. *japonica* JCM 21532^T^	81.2	XI	*P*. *japonica* JCM 21532^T^	91.9

^a^Strains FBF124 and FBF136 were included in phenotypic cluster VI-B and in group VI-C in the *rpoD* and MLSA analysis; strains FBF49, FBF82 and FBF91 were included in phenotypic cluster VI-F and in the *rpoD* gene group VI-B; strain FBF25 was included in phenotypic cluster I and in the *rpoD* gene and MLSA group IV.

### Phenotypic characterization and identification

The following preliminary phenotypic tests were used to identify the strains as possible members of the genus *Pseudomonas*: cell morphology, Gram staining, motility, oxidase, catalase, oxidation/fermentation of glucose and production of a fluorescent pigment on King B medium (S3 Table). All isolates were identified initially as *P*. *syringae* and *P*. *viridiflava* by the reactions in the LOPAT tests.

**Fig 2:**
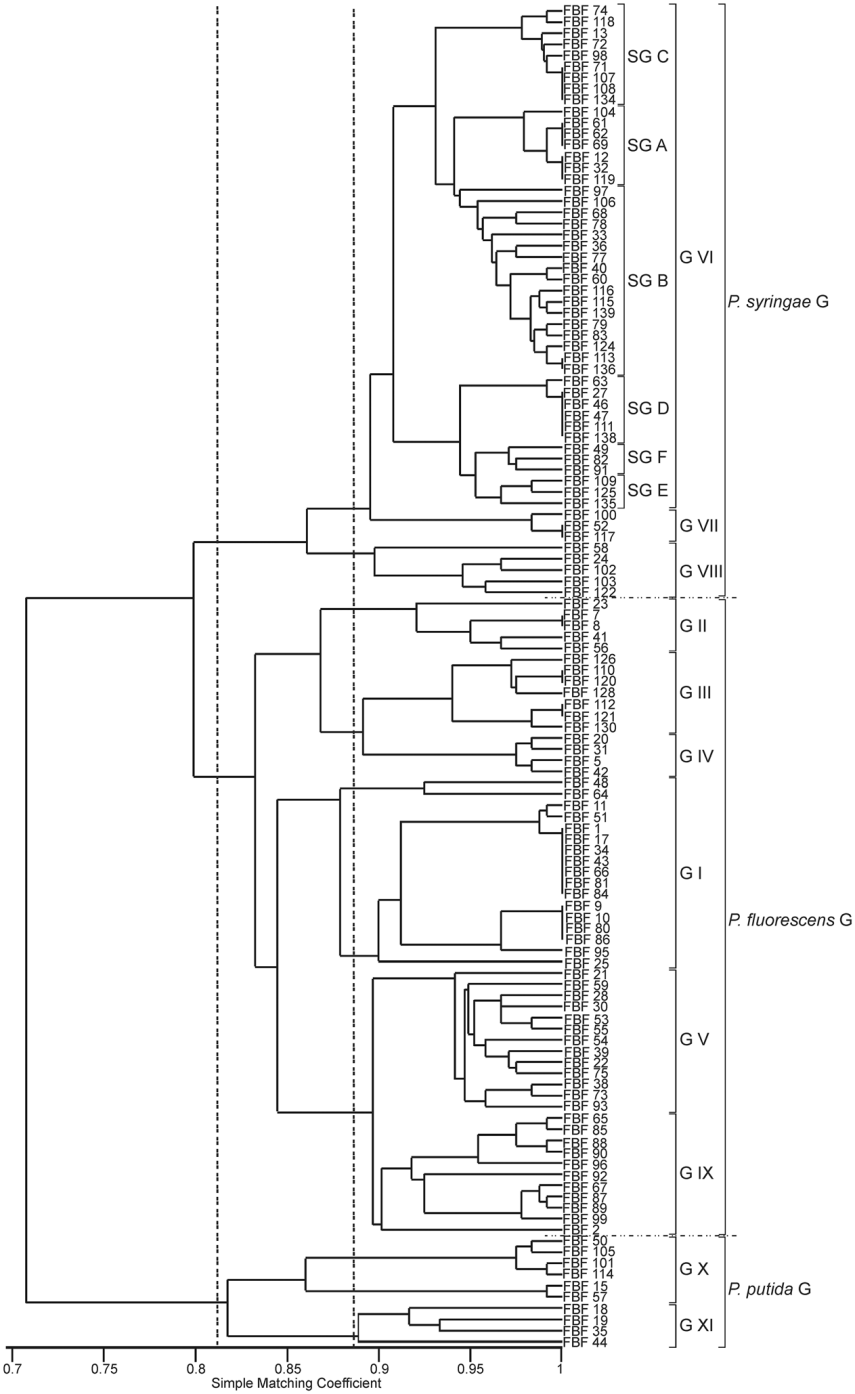
Phenotypic clustering of the studied strains. A similarity matrix based on the phenotypic traits was generated using a simple matching coefficient and the dendrogram constructed by UPGMA.

The results of the biochemical and physiological tests based on API 20 NE strips and Biolog GN2 microplates are given in S3 Table. A similarity matrix was generated using a simple matching coefficient, and the results are depicted in a dendrogram constructed by the UPGMA algorithm. Three main clusters were observed at 81% similarity, and these clusters corresponded to the *P*. *putida*, *P*. *syringae* and *P*. *fluorescens* phenogroups (Fig 2). Eleven phenotypic groupings were detected with 89–90% similarity; 2 clusters corresponded to the *P*. *putida* phenotype, 6 corresponded to the *P*. *fluorescens* phenotype, and 3 corresponded to the *P*. *syringae* phenotype. Exopolysaccharides (EPS) were only produced by strains belonging to the *P*. *fluorescens* phenotypic clusters I, II, and V and the *P*. *syringae* clusters VI and VII (Fig 2).

### The *rpoD* gene phylogenetic groups

**Fig 3:**
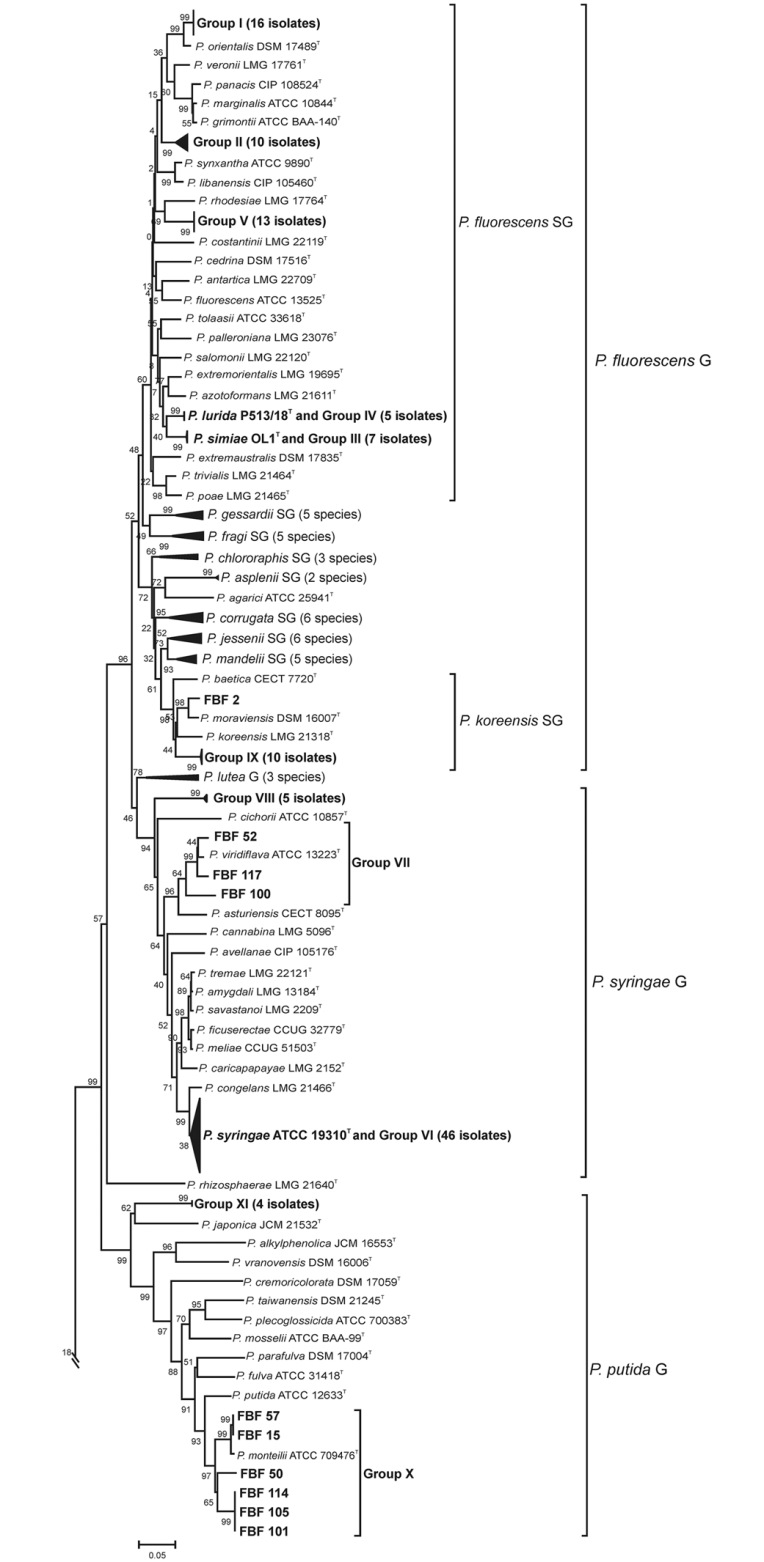
Phylogenetic tree of the *Pseudomonas* strains based on the nucleotide sequences of the *rpoD* gene (717 nt). The scale bar represents the number of substitutions per site. The number shown next to each node indicates the percentage bootstrap values of 1000 replicates. *Cellvibrio japonicus* was used as the outgroup.

In the majority of cases, the similarity of the isolates to the species type strains and the branching order in the *rpoD* phylogenetic tree (Fig 3 and Table 1) allowed for the identification of the strains at the species level. Eleven *rpoD* gene sequence groups were identified (Table 2). Forty-six isolates belonged to phenotypic cluster VI in the *P*. *syringae* species complex. Three isolates of phenotypic cluster VII were close to the *P*. *viridiflava* type strain. However, the remaining isolates were related to other *Pseudomonas* species. Five strains of phenotypic cluster VIII were located in an independent branch between the phylogenetic groups of *P*. *syringae* and *P*. *lutea*. Sixteen strains of phenotypic cluster I were located in the *P*. *fluorescens* phylogenetic subgroup, close to *P*. *orientalis*. The remaining isolates in the *P*. *fluorescens* phylogenetic subgroup clustered as follows: 7 strains of phenotypic cluster III were close to *P*. *simiae*; 5 strains of phenotypic cluster IV were close to *P*. *lurida*, 13 strains of phenotypic cluster V were close to *P*. *rhodesiae*; 11 strains of phenotypic cluster IX were close to *P*. *koreensis* and *P*. *moraviensis*; 10 strains of phenotypic cluster II formed an independent branch in the *P*. *fluorescens* phylogenetic subgroup, close to *P*. *libanensis*, *P*. *synxantha* and *P*. *veronii*. Some isolates were affiliated with species from the *P*. *putida* phylogenetic group; 6 strains of phenotypic cluster X were placed close to *P*. *monteilii*, and 4 strains of phenotypic cluster XI were close to *P*. *japonica*.

**Table 2:** Assignation to pathovars of strains identified as members of the *P*. *syringae* phylogenetic group based on *rpoD* gene sequences.

Strain	*P*. *syringae* group closest pathovar reference strain	Similarity (%)
**FBF27, FBF47**	*P*. *syringae* PDDCC 3023^T^	100
**FBF16, FBF36, FBF40, FBF49, FBF60, FBF68, FBF78, FBF79, FBF82, FBF83, FBF97, FBF106, FBF113, FBF115, FBF116, FBF139**	*P*. *syringae* pv. *dysoxyli* N255	99.1–99.5
**FBF24, FBF46, FBF58, FBF102, FBF103, FBF111, FBF122, FBF138**	*P*. *syringae* pv. *lapsa* N2096 / *P*. *syringae* pv. *atrofaciens* N2612	83.5–100
**FBF13, FBF71, FBF72, FBF74, FBF98, FBF107, FBF108, FBF118, FBF124, FBF134, FBF136**	*P*. *syringae* pv. *papulans* N2848	99.1–99.7
**FBF12, FBF32, FBF33, FBF61, FBF62, FBF69, FBF77, FBF91, FBF104, FBF109, FBF119, FBF125, FBF135**	*P*. *syringae* pv. *solidagae* ICMP 16925	97.7–98.4
**FBF63**	*P*. *syringae* pv. *syringae*	100
**FBF52, FBF100, FBF117**	*P*. *viridiflava* PDDCC 2848 / *P*. *syringae* pv. *ribicola* N963	90.7–97.9

**Fig 4:**
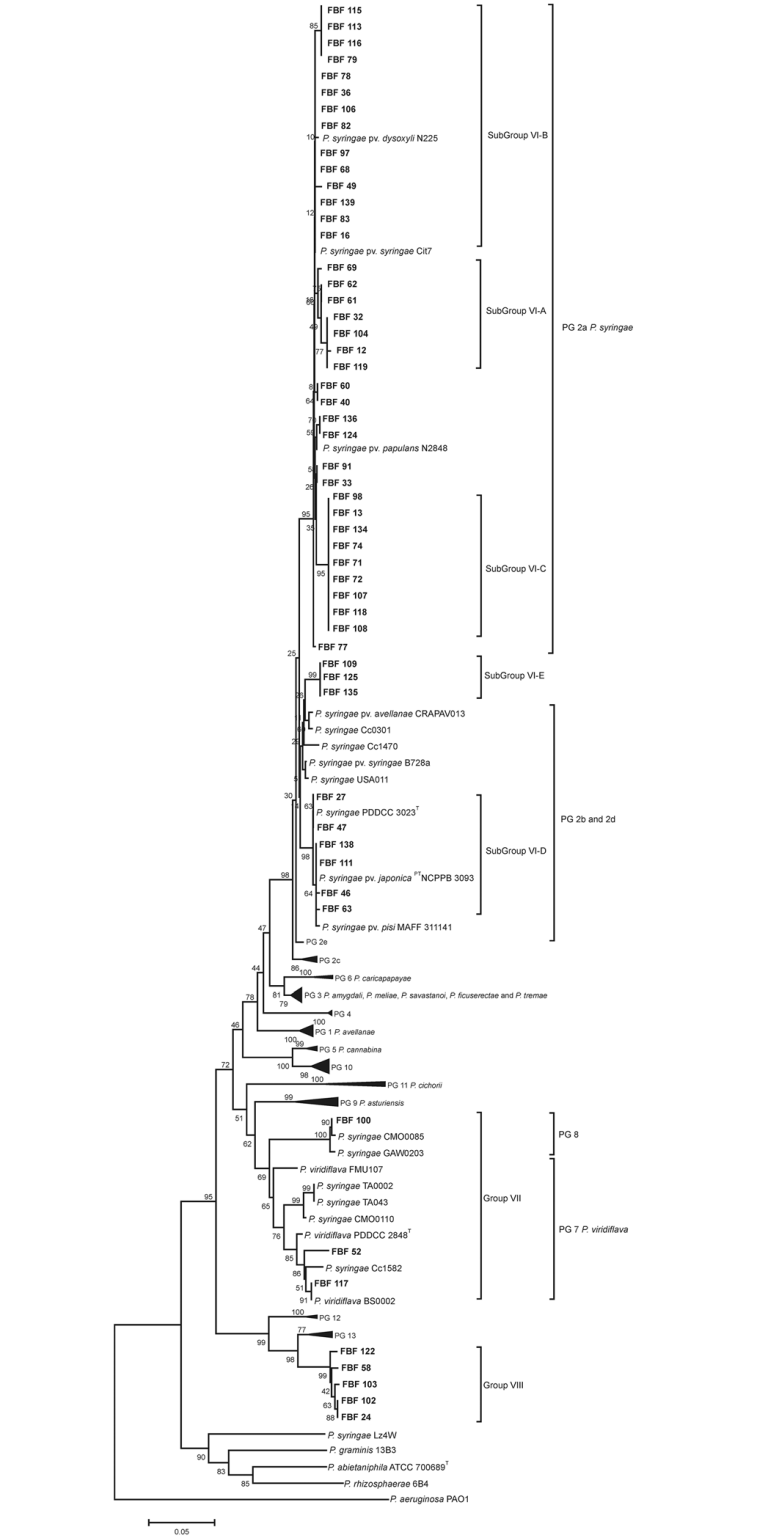
Phylogenetic tree based on the *rpoD* gene sequence (455 nt) of the strains assigned to the *P*. *syringae* species complex including the pathovar reference strains and strains of the 13 phylogroups (PG) defined by Berge et al. [26].

To further identify the 54 strains affiliated with the *P*. *syringae* species complex, a phylogenetic tree was constructed using the partial sequences of the *rpoD* gene (455 nt) published by Parkinson et al. [12] and Berge et al. [26]. As depicted in Fig 4 and Table 2, the majority of the strains in our study were closely affiliated in the *rpoD* sequence with strains of known pathovars of *P*. *syringae* (pv. *dysoxyli*, 16 strains; pv. *solidagae*, 10 strains; pv. *lapsa*/pv. *atrofaciens*, 3 strains; pv. *papulans*, 11 strains; and pv. *syringae*, 1 strain) and with strains classified in 3 of the 13 phylogroups (PG) defined by Berge and collaborators (PG 2a, 37 strains; PG 2b, 6 strains, together with *P*. *syringae* type strain; PG 2, but distinct to the subgroups already defined, 3 strains; PG7, 2 strains, together with *P*. *viridiflava* type strain) [26]. However, the 5 following strains appeared in a separate phylogroup in the species complex and were not affiliated with any known pathovar or phylogroup: strains FBF24, 58, 102, 103 and 122.

### Multilocus sequence analysis

**Fig 5:**
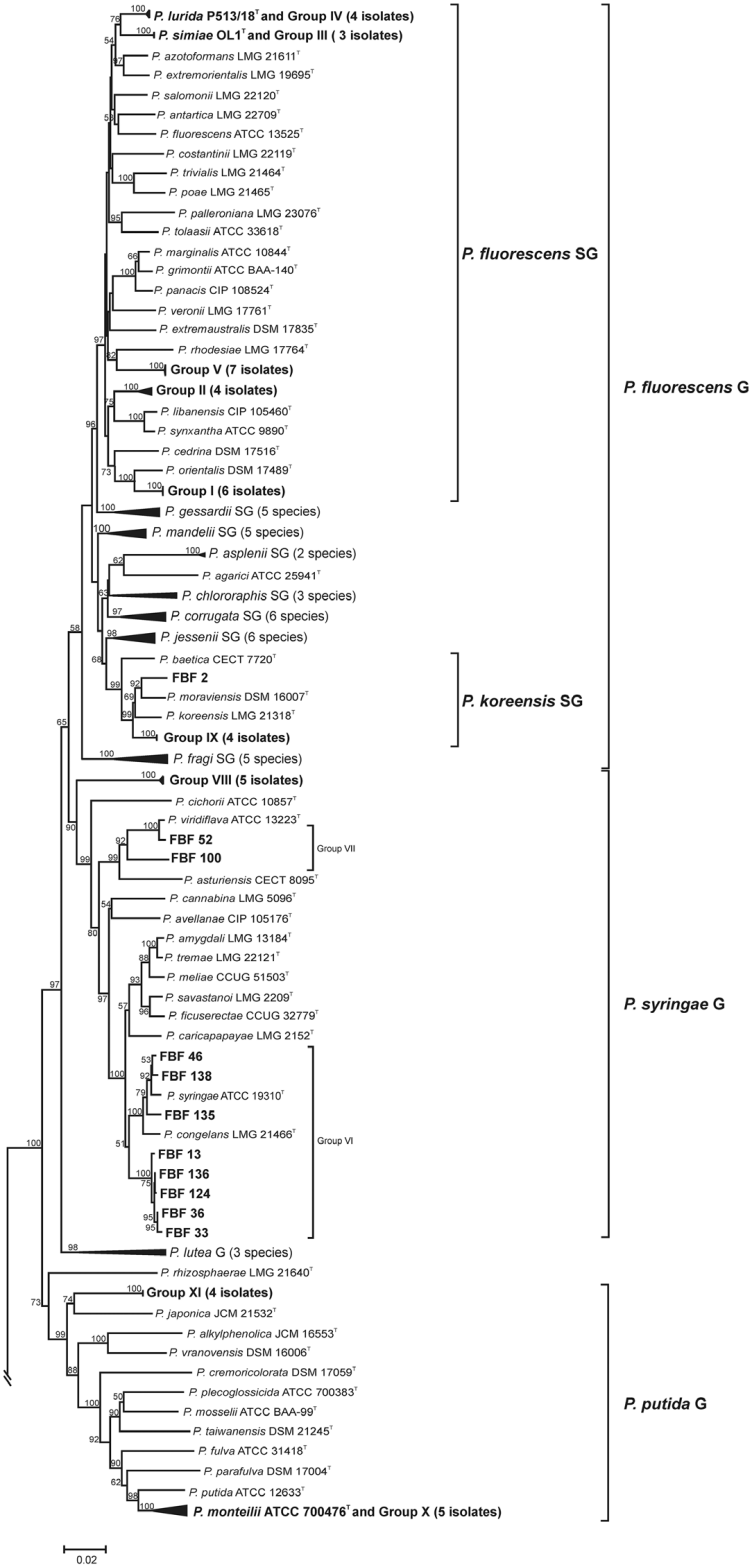
Phylogenetic consensus tree of the *Pseudomonas* strains based on the nucleotide sequences of the 16S rRNA, *rpoD*, and *gyrB* genes of 53 selected strains. The scale bar represents the number of substitutions per site. The number shown next to each node indicates the percentage bootstrap values of 1000 replicates. *Cellvibrio japonicus* was used as the outgroup.

To determine the phylogenetic affiliation of the *rpoD* gene groupings in the genus *Pseudomonas*, 53 strains were selected as representatives of the 11 *rpoD* gene groupings for a multilocus sequence analysis (MLSA) according to the following criterion: at least 2 strains per *rpoD* phylogenetic group were selected to have representative strains of each phylogroup and to assess the phylogenetic diversity within each group. Phylogenetic analyses based on the concatenated sequences (2,818 nt) of the 16S rRNA (1,296 nt), *gyrB* (805 nt) and *rpoD* genes (717 nt) confirmed the distribution of the isolates in the 11 phylogenetic and phenotypic groupings (Figs 2, 3 and 4). The robustness of the concatenated tree was demonstrated by the high bootstrap values at all branches. The MLSA groups (Fig 5) were congruent with the groups in the *rpoD* analysis. The 53 representative strains were located in the *P*. *fluorescens* phylogenetic group (5 in the *P*. *koreensis* SG; 24 in the *P*. *fluorescens* SG), the *P*. *syringae* group (15 strains), and the *P*. *putida* group (9 strains) (Fig 5).

### Assignment of the strains to species

Following the criteria proposed by Mulet and collaborators [5], the strains were assigned to known species in the MLSA analysis when the similarity to a type strain was higher than 97% and both strains were located in the same phylogenetic branch (Fig 5). Assignments are indicated in Table 1 and S2 Table. Group VI was the largest among the 11 groups, and the eight strains comprising the group were members of the *P*. *syringae* group. These eight strains were located in two independent branches; one branch had three isolates that were close to *P*. *syringae* and *P*. *congelans*, and there were five strains in the other independent branch. Group VII (two strains) consisted of isolates identified as *P*. *viridiflava*. Group VIII (5 strains) included isolates that have a similarity lower than 92% with the type strain of any other species in the group and are, therefore, considered members of a putative new species in the *P*. *syringae* phylogenetic group (Fig 5).

The other 6 groups (I, II, III, IV, V and IX) are affiliated with the *P*. *fluorescens* phylogenetic group as defined by Mulet and collaborators [27, 5] and are closely related to species that have not previously been described as plant pathogens. The highest similarity detected for the isolates of Group I was with *P*. *orientalis* (96.9–97.0%). The isolates of Group II were close to the type strains of *P*. *libanensis* and *P*. *synxantha* (95.9–96.0% for isolate FBF23; 95.9–96.0% for isolate FBF56).

The 3 strains of Group III are phylogenetically close to *P*. *simiae* (99.9%). The 4 strains of Group IV are phylogenetically close to *P*. *lurida* (99.7% for strains FBF25, 31 and 42 and 97.7% for strain FBF5). The 7 isolates of Group V showed a similarity of 94.8–94.9% with *P*. *marginalis* and *P*. *grimontii*. The isolates of Group IX are related to the two species of the *P*. *koreensis* subgroup, *P*. *koreensis* (96.7% similarity) and *P*. *moraviensis* (96.8% similarity).

Members of the two remaining groups (X and XI) belonged to the *P*. *putida* phylogenetic group. The isolates of Group X are related to *P*. *monteilii* (95.8–98.9% similarity), which is a species isolated from clinical specimens [28] and environmental samples [29]. The closest type strain to the isolates of Group XI was *P*. *japonica*, although this strain exhibited a low level of similarity (91.9%).

The distribution of the strains in groups and the geographical regions of isolation are indicated in S1 and S2 Tables and in S1 Fig. Representative strains of all groups were detected in the Mazandaran region, groups II, III, IV, VII, VIII and X were not detected in the Gilan region and groups II, V, VII and XI were not detected in the Golestan region; groups I, VI and IX were present in all 3 regions. There was no correlation between cultivars and groups. Strains representatives of all groups were isolated in more than one cultivar.

## Discussion

The physiological and biochemical characteristics of the isolates were consistent with the characteristics previously described for the genus *Pseudomonas*, although discrepancies with known features of the classical citrus pathogens existed in the LOPAT tests (levan production, oxidase activity, potato soft rot, arginine dihydrolase activity, tobacco hypersensitivity). The phytopathogenic, oxidase-negative fluorescent *Pseudomonas* species have been traditionally identified as either *P*. *syringae* or *P*. *viridiflava*. Several strains in our study (71 strains) showed a LOPAT pattern different from the patterns corresponding to *P*. *syringae* or *P*. *viridiflava*. The polyphasic taxonomic study of the 126 strains analyzed demonstrated that only strains in group VI (*P*. *syringae*, 46 strains) and group VII (*P*. *viridiflava*, 2 strains) could be identified as members of these two species. Strains belonging to *P*. *orientalis*, *P*. *synxantha*, *P*. *simiae*, *P*. *lurida*, and *P*. *monteilii* were also identified; furthermore, at least 5 putative novel species were identified.

The molecular data confirmed that the partial sequence analysis of the *rpoD* gene is sufficient for rapid isolate classification. The same strain groupings were obtained using the *rpoD* sequence alone and in the analysis based on the concatenated 3 genes (16S rRNA, *gyrB* and *rpoD*), as was previously described for *Pseudomonas* species [27] and for the pathovars in *P*. *syringae* [12]. The MLSA phylogroups defined by Parkinson et al. [12] and Berge et al. [26] are maintained in this study. The groupings of strains obtained by the molecular methods in the MLSA study were similar to the groupings obtained by the biochemical and physiological tests. The combined results showed a high diversity among *Pseudomonas* strains pathogenic to citrus in Iran. No clear distribution of species by region was detected, and only members of group I assigned to *P*. *orientalis*, group VI assigned to *P*. *syringae* and group IX that could not be assigned to a known species were found in the three geographical regions. The severity of the lesions induced by the strains assigned to species not yet described as pathogenic and the putative novel species were of a similar order of magnitude as the *P*. *syringae* and *P*. *viridiflava* isolates.

Plant pathogenic *Pseudomonas* spp. have been described in the *P*. *fluorescens* phylogenetic lineage rather than in the *P*. *aeruginosa* lineage [16]. The 21 *Pseudomonas* species described by Bull and collaborators [16] belong mainly to the *P*. *syringae* phylogenetic group (14 species), with 5 species associated with the *P*. *fluorescens* subgroup and 2 species associated with the *P*. *corrugata* subgroup. The majority of the strains used in the present study belonged to the *P*. *syringae* phylogenetic group (54 strains) and the *P*. *fluorescens* subgroup (51 strains). Several strains were assigned to species that have not been described as plant pathogens and have been isolated previously from diverse habitats. *P*. *orientalis* (group I, 16 strains) has been isolated from spring waters in Lebanon [30]; *P*. *simiae* (group III, 7 strains) has been isolated from a monkey [31] and from Antarctic samples [32]; *P*. *lurida* (group IV, 5 strains) has been found as a plant growth promoting bacterium in the phyllosphere of grasses [33] and in high altitude rhizospheric soil from the Uttarakh and Himalayas [34, 35]; *P*. *monteilii* (group X, 6 strains) has been isolated from clinical specimens [28] and environmental samples [29]. Strains in groups II (10 strains), V (13 strains), VII (1 strain), VIII (5 strains), IX (10 strains), X (4 strains) and XI (4 strains), which represented 37% of the isolates, could not be assigned to a known species and are considered representatives of at least 5 putative novel *Pseudomonas* species because each group is phylogenetically and phenotypically homogeneous and distinct from known species type strains.

The results of the present study showed that citrus blast disease in the northern citrus producing provinces is caused by a diverse population of *Pseudomonas* strains belonging to species and pathovars including the two universally known species *P*. *syringae* pv. *syringae* [36] and *P*. *viridiflava* [7]. We demonstrated, for the first time, that the *P*. *lurida*, *P*. *orientalis* and *P*. *simiae* strains are pathogenic to plants. The probable differences between these species in characteristics such as host range with and outside *Rutaceae* and over summering are new issues in need of resolution. The analysis of the *rpoD* gene partial sequences is a fast and reliable method to differentiate *Pseudomonas* plant pathogenic species, and the *Pseudomonas* species diversity is higher than previously thought for citrus plants. At least 5 putative novel species have been detected, and their formal proposal is underway. These results are in accordance with the proposal of Scotta and collaborators [37], who recommended the use of the *rpoD* gene for routine and epidemiological studies of *Pseudomonas* clinical strains. This method is well suited for routine assessments of citrus plant lots for quality control to limit the chance of increasing the genetic diversity of *Pseudomonas* populations through the importation of foreign plants.

## Supporting Information

S1 Fig Distribution per geographical region of the 11 groups of strains in this study. (PDF)

S1 Table Sequence accession numbers, geographical origins, *Citrus* hosts and sampling dates of the strains used in this study. (PDF)

S2 Table List of the strains used in this study and their assignation to phenotypic clusters and phylogenetic groups in the *rpoD* gene tree and in the MLSA analysis. (PDF)

S3 Table Phenotypic characterization and pathogenicity tests of the strains included in this study. ++, strongly positive; +, positive; w+, weakly positive; -, negative; ND, not determined.
(XLSX)
