# Supplementary figures and images for: New Pseudomonas spp. Are Pathogenic to Citrus
Source: PLoS One. 2016 Feb 26;11(2):e0148796. doi: 10.1371/journal.pone.0148796 (PMC4769151; doi:10.1371/journal.pone.0148796)

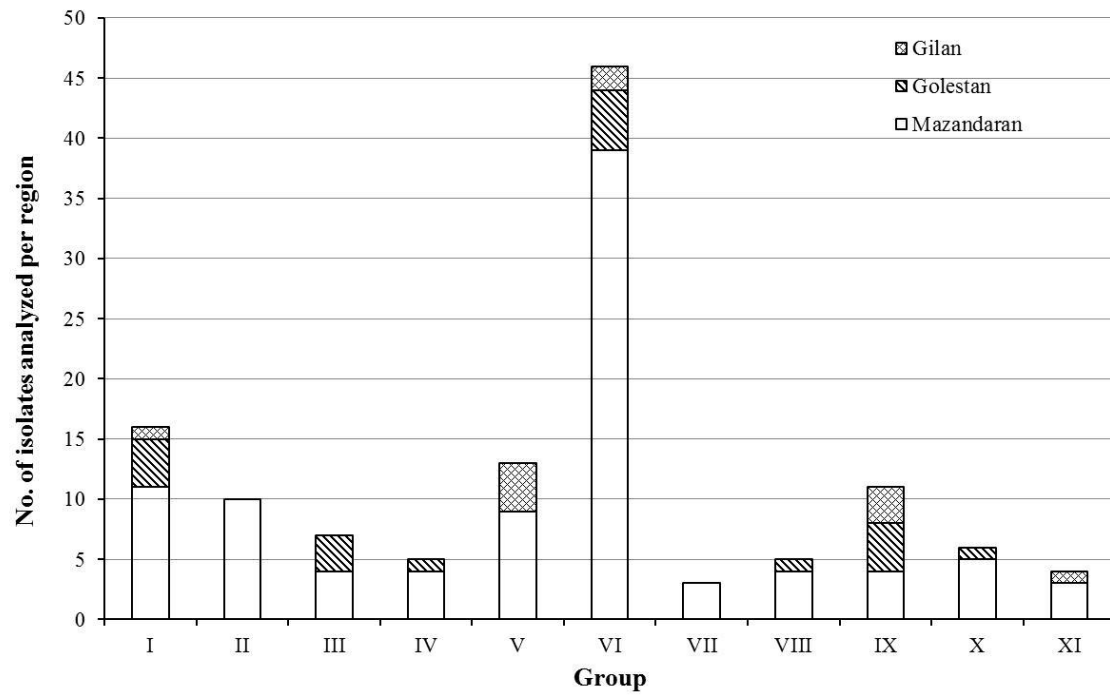

**S1 Fig.** Distribution per geographical region of the 11 groups of strains in this study.

Supplement: S1 Fig — (PDF) [file pone.0148796.s001.pdf]
