# Supplementary material for: New Pseudomonas spp. Are Pathogenic to Citrus
Source: PLoS One. 2016 Feb 26;11(2):e0148796. doi: 10.1371/journal.pone.0148796 (PMC4769151; doi:10.1371/journal.pone.0148796)
Supplement: S1 Table — (PDF) [file pone.0148796.s002.pdf]

S1 Table. Sequence accession numbers, geographical origins, C*itrus* hosts and sampling dates of the strains used in this study.

Strain	Accession Nr 16S rRNA gene	Accession Nr *rpoD* gene	Accession Nr *gyrB* gene	Province	City	GPS position	Host	Year of isolation
FBF1	HG805683	HG805502	HG805628	Mazandaran	Fereydun knar	N36 40.957 E52 30.419	Sour Orange	2009
FBF2	HG805684	HG805503	HG805629	Mazandaran	Babol	N36 31.378 E52 39.287	Washington Navel	2009
FBF5	HG805685	HG805504	HG805630	Mazandaran	Juybar	N36 40.873 E52 54.422	Washington Navel	2009
FBF7	HG805686	HG805505	HG805631	Mazandaran	Amol	N36 31.862 E52 31.900	Sour Orange	2009
FBF8	HG805687	HG805506	HG805632	Mazandaran	Amol	N36 31.862 E52 31.900	Sour Orange	2009
FBF9	HG805688	HG805507	nd	Mazandaran	Chamestan	N36 26.313 E52 13.572	Local Orange	2009
FBF10	HG805689	HG805508	nd	Mazandaran	Babol Bandpei	N36 29.481 E52 39.950	Local Orange	2009
FBF11	HG805690	HG805509	nd	Mazandaran	Babol Bandpei	N36 29.481 E52 39.950	Local Orange	2009
FBF12	HG805691	HG805510	nd	Mazandaran	Babol Bandpei	N36 22.833 E52 40.121	Local Orange	2009
FBF13	HG805692	HG805511	HG805633	Mazandaran	Babol Bandpei	N36 21.108 E52 38.989	Local Orange	2009
FBF15	HG805693	HG805512	nd	Mazandaran	Qaemshahr	N36 31.677 E52 48.954	Local Orange	2009
FBF16	HG805694	HG805513	nd	Mazandaran	Ghaemshahr	N36 31.677 E52 48.954	Local Orange	2009
FBF17	HG805695	HG805514	nd	Mazandaran	Chalus	N36 37.577 E51 32.143	Local Orange	2009
FBF18	HG805696	HG805515	HG805634	Mazandaran	Chalus	N36 38.185 E51 28.737	Local Orange	2009
FBF19	HG805697	HG805516	HG805635	Mazandaran	Nashtaroud	N36 44.009 E51 00.827	Local Orange	2009
FBF20	HG805698	HG805517	nd	Mazandaran	Nashtaroud	N36 44.009 E51 00.827	Local Orange	2009
FBF21	HG805699	HG805518	HG805636	Gilan	Holosara	N37 02.530 E50 15.891	Citrange	2009
FBF22	HG805700	HG805519	nd	Mazandaran	Sari	N36 43.644 E52 59.544	Washington Navel	2009
FBF23	HG805701	HG805520	HG805637	Mazandaran	Babol	N36 22.665 E52 40.282	Washington Navel	2010
FBF24	HG805702	HG805521	HG805638	Mazandaran	Babol	N36 22.749 E52 39.648	Washington Navel	2010
FBF25	HG805703	HG805522	HG805639	Mazandaran	Babol	N36 19.736 E52 38.403	Washington Navel	2010
FBF27	HG805704	HG805523	nd	Mazandaran	Babol	N36 22.833 E52 40.121	Washington Navel	2010
FBF28	HG805705	HG805524	nd	Mazandaran	Babol	N36 24.432 E52 40.313	Washington Navel	2010

FBF30	HG805706	HG805525	HG805640	Mazandaran	Sari	N36 31.000	E52 57.798	Unshiu	2010
FBF31	HG805707	HG805526	HG805641	Golestan	Galogah	N36 44.558	E53 52.727	Sour Orange	2010
FBF32	HG805708	HG805527	nd	Golestan	Gorgan	N36 51.108	E54 31.278	Sour Orange	2010
FBF33	HG805709	HG805528	HG805642	Golestan	Gorgan	N36 50.695	E54 23.890	Sour Orange	2010
FBF34	HG805710	HG805529	nd	Gilan	Roudsar	N37 04.304	E50 21.767	Washington Navel	2010
FBF35	HG805711	HG805530	HG805643	Gilan	Amlash	N37 02.374	E50 18.407	Local Orange	2010
FBF36	HG805712	HG805531	HG805644	Gilan	Langroud	N37 10.278	E50 07.923	Sour Orange	2010
FBF38	HG805713	HG805532	HG805645	Mazandaran	Kela chay	N36 59.168	E50 33.271	Sour Orange	2010
FBF39	HG805714	HG805533	nd	Mazandaran	Ramsar	N36 55.470	E50 40.108	Sour Orange	2010
FBF40	HG805715	HG805534	nd	Mazandaran	Ramsar	N36 55.470	E50 40.108	Sour Orange	2010
FBF41	HG805716	HG805535	nd	Mazandaran	Tonekabon	N36 45.767	E50 52.871	Sour Orange	2010
FBF42	HG805717	HG805536	HG805646	Mazandaran	Chalus	N36 37.579	E51 32.158	Washington Navel	2010
FBF43	HG805718	HG805537	nd	Mazandaran	Mahmod abad	N36 38.499	E52 17.465	Sour Orange	2010
FBF44	HG805719	HG805538	HG805647	Mazandaran	Chamestan	N36 27.297	E52 14.180	Sour Orange	2010
FBF46	HG805720	HG805539	HG805648	Mazandaran	Babol	N36 29.566	E52 34.069	Washington Navel	2010
FBF47	HG805721	HG805540	nd	Mazandaran	Babol	N36 29.566	E52 34.069	Sour Orange	2010
FBF48	HG805722	HG805541	HG805649	Mazandaran	Bahnemir	N36 37.389	E52 42.945	Sour Orange	2010
FBF49	HG805723	HG805542	nd	Mazandaran	Behshahr	N36 43.677	E53 45.820	Washington Navel	2010
FBF50	HG805724	HG805543	HG805650	Mazandaran	Sari	N36 43.644	E52 59.544	Washington Navel	2009
FBF51	HG805725	HG805544	HG805651	Mazandaran	Juybar	N36 35.237	E52 49.900	Washington Navel	2009
FBF52	HG805726	HG805545	HG805652	Mazandaran	Chalus	N36 38.185	E51 28.737	Local Orange	2009
FBF53	HG805727	HG805546	nd	Gilan	Holosara	N37 02.530	E50 15.891	Citrange	2009
FBF54	HG805728	HG805547	HG805653	Gilan	Holosara	N37 02.530	E50 15.891	Citrange	2009
FBF55	HG805729	HG805548	HG805654	Gilan	Holosara	N37 02.530	E50 15.891	Citrange	2009
FBF56	HG805730	HG805549	HG805655	Mazandaran	Ramsar	N36 52.568	E50 45.632	Washington Navel	2009
FBF57	HG805731	HG805550	HG805656	Mazandaran	Behshahr	N36 42.613	E53 40.277	Sour Orange	2009
FBF58	HG805732	HG805551	HG805657	Mazandaran	Chalus	N36 41.144	E51 20.702	Sour Orange	2009
FBF59	HG805733	HG805552	HG805658	Mazandaran	Babol	N36 29.566	E52 34.069	Washington Navel	2010

FBF60	HG805734	HG805553	nd	Mazandaran	Babol	N36 21.143	E52 38.914	Washington Navel	2010
FBF61	HG805735	HG805554	nd	Mazandaran	Amol	N36 30.092	E52 27.216	Sour Orange	2010
FBF62	HG805736	HG805555	nd	Mazandaran	Zargarmahale	N36 32.921	E52 32.725	Sour Orange	2010
FBF63	HG805737	HG805556	nd	Mazandaran	Zargarmahale	N36 33.827	E52 33.706	Sour Orange	2010
FBF64	HG805738	HG805557	HG805659	Mazandaran	Zargarmahale	N36 34.224	E52 35.154	Sour Orange	2010
FBF65	HG805739	HG805558	HG805660	Mazandaran	Zargarmahale	N36 35.379	E52 35.489	Sour Orange	2010
FBF66	HG805740	HG805559	nd	Mazandaran	Kalebast	N36 37.856	E52 36.995	Sour Orange	2010
FBF67	HG805741	HG805560	nd	Mazandaran	Amirkola	N36 36.281	E52 40.569	Sour Orange	2010
FBF68	HG805742	HG805561	nd	Mazandaran	Amirkola	N36 35.671	E52 40.662	Sour Orange	2010
FBF69	HG805743	HG805562	nd	Mazandaran	Juybar	N36 32.946	E52 40.781	Sour Orange	2010
FBF71	HG805744	HG805563	nd	Mazandaran	Juybar	N36 36.099	E52 43.048	Washington Navel	2010
FBF72	HG805745	HG805564	HG805661	Mazandaran	Bahnemir	N36 39.494	E52 45.597	Washington Navel	2010
FBF73	HG805746	HG805565	nd	Mazandaran	Bahnemir	N36 39.494	E52 45.597	Washington Navel	2010
FBF74	HG805747	HG805566	nd	Mazandaran	Bahnemir	N36 39.494	E52 45.597	Washington Navel	2010
FBF75	HG805748	HG805567	nd	Mazandaran	Bahnemir	N36 39.494	E52 45.597	Washington Navel	2010
FBF77	HG805749	HG805568	nd	Mazandaran	Sari	N36 41.096	E53 03.822	Washington Navel	2010
FBF78	HG805750	HG805569	nd	Mazandaran	Sari	N36 31.000	E52 57.798	Unshiu	2010
FBF79	HG805751	HG805570	nd	Mazandaran	Sari	N36 31.000	E52 57.798	Local Orange	2010
FBF80	HG805752	HG805571	nd	Golestan	Galogah	N36 44.558	E53 52.727	Sour Orange	2010
FBF81	HG805753	HG805572	nd	Golestan	Galogah	N36 44.558	E53 52.727	Sour Orange	2010
FBF82	HG805754	HG805573	nd	Golestan	Galogah	N36 44.558	E53 52.727	Sour Orange	2010
FBF83	HG805755	HG805574	nd	Golestan	Gorgan	N36 51.108	E54 31.278	Sour Orange	2010
FBF84	HG805756	HG805575	HG805662	Golestan	Gorgan	N36 50.695	E54 23.890	Sour Orange	2010
FBF85	HG805757	HG805576	nd	Golestan	Kordkuy	N36 47.503	E54 05.000	Sour Orange	2010
FBF86	HG805758	HG805577	HG805663	Golestan	Bandar gaz	N36 45.203	E53 55.359	Sour Orange	2010
FBF87	HG805759	HG805578	nd	Golestan	Bandar gaz	N36 45.203	E53 55.359	Sour Orange	2010
FBF88	HG805760	HG805579	nd	Golestan	Bandar gaz	N36 45.193	E53 55.362	Washington Navel	2010
FBF89	HG805761	HG805580	nd	Gilan	Roudsar	N37 04.304	E50 21.767	Washington Navel	2010

FBF90	HG805762	HG805581	nd	Gilan	Hajiabad	N37 02.374	E50 18.407	Local Orange	2010
FBF91	HG805763	HG805582	HG805664	Gilan	Garmabsara	N37 03.081	E50 16.029	Sour Orange	2010
FBF92	HG805764	HG805583	HG805665	Gilan	Langroud	N37 10.278	E50 07.923	Sour Orange	2010
FBF93	HG805765	HG805584	HG805666	Mazandaran	Ramsar	N36 55.470	E50 40.108	Sour Orange	2010
FBF95	HG805766	HG805585	nd	Mazandaran	Chalus	N36 37.579	E51 32.158	Washington Navel	2010
FBF96	HG805767	HG805586	HG805667	Mazandaran	Amol	N36 30.578	E52 20.726	Sour Orange	2010
FBF97	HG805768	HG805587	nd	Mazandaran	Bahnemir	N36 39.494	E52 45.597	Washington Navel	2010
FBF98	HG805769	HG805588	nd	Mazandaran	Juybar	N36 42.347	E52 55.543	Sour Orange	2010
FBF99	HG805770	HG805589	HG805668	Golestan	Kordkuy	N36 45.785	E53 59.275	Sour Orange	2010
FBF100	HG805771	HG805590	HG805669	Mazandaran	Babol	N36 28.835	E52 39.746	Washington Navel	2009
FBF101	HG805772	HG805591	HG805670	Golestan	Bandar gaz	N36 46.145	E54 00.446	Washington Navel	2009
FBF102	HG805773	HG805592	HG805671	Mazandaran	Babol	N36 22.749	E52 39.648	Washington Navel	2010
FBF103	HG805774	HG805593	HG805672	Golestan	Bandar gaz	N36 45.203	E53 55.359	Sour Orange	2010
FBF104	HG805775	HG805594	nd	Mazandaran	Kela chay	N37 02.632	E50 27.448	Sour Orange	2010
FBF105	HG805776	HG805595	HG805673	Mazandaran	Chalus	N36 37.579	E51 32.158	Washington Navel	2010
FBF106	HG805777	HG805596	nd	Mazandaran	Mahmod abad	N36 38.499	E52 17.465	Sweet lime	2010
FBF107	HG805778	HG805597	nd	Mazandaran	Mahmod abad	N36 38.499	E52 17.465	Sour Orange	2010
FBF108	HG805779	HG805598	nd	Mazandaran	Mahmod abad	N36 38.499	E52 17.465	Sour Orange	2010
FBF109	HG805780	HG805599	nd	Mazandaran	Izdeh	N36 34.633	E52 01.859	Local Orange	2010
FBF110	HG805781	HG805600	HG805674	Mazandaran	Nour	N36 29.988	E52 04.911	Sour Orange	2010
FBF111	HG805782	HG805601	nd	Mazandaran	Chamestan	N36 28.836	E52 13.182	Sour Orange	2010
FBF112	HG805783	HG805602	nd	Mazandaran	Chamestan	N36 27.297	E52 14.180	Sour Orange	2010
FBF113	HG805784	HG805603	nd	Mazandaran	Amol	N36 27.297	E52 14.180	Sour Orange	2010
FBF114	HG805785	HG805604	HG805675	Mazandaran	Amol	N36 30.578	E52 20.726	Sour Orange	2010
FBF115	HG805786	HG805605	nd	Mazandaran	Babol	N36 36.099	E52 43.048	Washington Navel	2010
FBF116	HG805787	HG805606	nd	Mazandaran	Bahnemir	N36 37.389	E52 42.945	Sour Orange	2010
FBF117	HG805788	HG805607	nd	Mazandaran	Juybar	N36 41.328	E52 52.256	Sour Orange	2010
FBF118	HG805789	HG805608	nd	Mazandaran	Juybar	N36 42.347	E52 55.543	Sour Orange	2010

FBF119	HG805790	HG805609	nd	Golestan	Gorgan	N36 51.108 E54 31.278	Sour Orange	2010
FBF120	HG805791	HG805610	nd	Golestan	Gorgan	N36 51.108 E54 31.278	Sour Orange	2010
FBF121	HG805792	HG805611	nd	Golestan	Bandar gaz	N36 45.203 E53 55.359	Sour Orange	2010
FBF122	HG805793	HG805612	HG805676	Mazandaran	Nashtaroud	N36 44.157 E51 01.020	Local Orange	2009
FBF124	HG805794	HG805613	HG805677	Mazandaran	Babol	N36 22.749 E52 39.648	Washington Navel	2010
FBF125	HG805795	HG805614	nd	Mazandaran	Babol	N36 21.118 E52 39.004	Washington Navel	2010
FBF126	HG805796	HG805615	nd	Mazandaran	Chaboksar	N36 58.489 E50 34.721	Page	2010
FBF128	HG805797	HG805616	HG805678	Mazandaran	Tonekabon	N36 42.922 E50 58.633	Sour Orange	2010
FBF130	HG805798	HG805617	HG805679	Golestan	Gorgan	N36 50.695 E54 23.890	Sour Orange	2010
FBF134	HG805799	HG805618	nd	Mazandaran	Babol	N36 29.566 E52 34.069	Washington Navel	2010
FBF135	HG805800	HG805620	HG805680	Mazandaran	Babol	N36 22.749 E52 39.648	Washington Navel	2010
FBF136	HG805801	HG805619	HG805681	Mazandaran	Mahmod abad	N36 38.499 E52 17.465	Sour Orange	2010
FBF138	HG805802	HG805621	HG805682	Mazandaran	Nour	N36 34.633 E52 01.859	Sour Orange	2010
FBF139	HG805803	HG805622	nd	Mazandaran	Chamestan	N36 29.988 E52 04.911	Sour Orange	2010
FBF140	HG805804	HG805623	nd	Mazandaran	Chamestan	N36 29.988 E52 04.911	Sour Orange	2010
FBF141	HG805805	HG805624	nd	Mazandaran	Chamestan	N36 28.836 E52 13.182	Sour Orange	2010
FBF142	HG805806	HG805625	nd	Mazandaran	Chamestan	N36 27.297 E52 14.180	Sour Orange	2010
FBF143	HG805807	HG805626	nd	Mazandaran	Chamestan	N36 27.297 E52 14.180	Sour Orange	2010
FBF144	HG805808	HG805627	nd	Mazandaran	Amol	N36 30.578 E52 20.726	Sour Orange	2010

nd, not determined
