# Supplementary material for: New Pseudomonas spp. Are Pathogenic to Citrus
Source: PLoS One. 2016 Feb 26;11(2):e0148796. doi: 10.1371/journal.pone.0148796 (PMC4769151; doi:10.1371/journal.pone.0148796)
Supplement: S2 Table — (PDF) [file pone.0148796.s003.pdf]

S2 Table. List of the strains used in this study and their assignation to phenotypic clusters and phylogenetic groups in the *rpoD* gene tree and in the MLSA analysis.

Strain	Phenotypic cluster	*rpoD* group	*rpoD* closest type strain	Similarity (%)	Phylogenetic group (3 genes)	Closest type strain (3 genes)	Similarity (%)	Species assignation (97% 3 genes)
FBF1	I	I	*P. orientalis* DSM 17489^T	97.8	I	*P. orientalis* DSM 17489^T	97	*P. orientalis*
FBF2	IX	IX	*P. moraviensis* DSM 16007^T	97.2	IX	*P. moraviensis* DSM 16007^T	97.2	*P. moraviensis*
FBF5	IV	IV	*P. lurida* P513/18^T	99.5	IV	*P. lurida* P513/18^T	99.6	*P. lurida*
FBF7	II	II	*P. synxantha* LMG 2335^T	93.3	II	*P. synxantha* LMG 2335^T	96	Putative new species
FBF8	II	II	*P. synxantha* LMG 2335^T	93.7	II	*P. synxantha* LMG 2335^T	96	Putative new species
FBF9	I	I	*P. orientalis* DSM 17489^T	97.8	nd			*P. orientalis*
FBF10	I	I	*P. orientalis* DSM 17489^T	97.8	nd			*P. orientalis*
FBF11	I	I	*P. orientalis* DSM 17489^T	97.8	nd			*P. orientalis*
FBF12	VI-A	VI-A	*P. syringae* ATCC 19310^T	97.4	nd			*P. syringae*
FBF13	VI-C	VI-C	*P. syringae* ATCC 19310^T	97.5	VI	*P. tremae* LMG 22121^T / *P. syringae* ATCC 19310^T	97.1	*P. tremae*
FBF15	X	X	*P. monteilii* ATCC 700476^T	99	nd			*P. monteilii*
FBF16	nd	VI-B	*P. syringae* ATCC 19310^T	98.2	nd			*P. tremae*
FBF17	I	I	*P. orientalis* DSM 17489^T	97.8	nd			*P. orientalis*
FBF18	XI	XI	*P. japonica* JCM 21532^T	81.2	XI	*P. japonica* JCM 21532^T	91.9	Putative new species
FBF19	XI	XI	*P. japonica* JCM 21532^T	81.2	XI	*P. japonica* JCM 21532^T	91.9	Putative new species
FBF20	IV	IV	*P. lurida* P513/18^T	99.5	nd			*P. lurida*
FBF21	V	V	*P. rhodesiae* LMG 17764^T	91.7	V	*P. marginalis* ATCC 10844^T / *P. grimontii* CIP 106645^T	94.8	Putative new species

FBF22	V	V	*P. rhodesiae* LMG 17764^T	91.7	nd			Putative new species
FBF23	II	II	*P. libanensis* CIP105460^T	93.7	II	*P. synxantha* LMG 2335^T	96	Putative new species
FBF24	VIII	VIII	*P. syringae* ATCC 19310^T	85.2	VIII	*P. meliae* CCUG 51503^T	91.6	Putative new species
FBF25	I	IV	*P. lurida* P513/18^T	99.5	IV	*P. lurida* P513/18^T	99.7	*P. lurida*
FBF27	VI-D	VI-D	*P. syringae* ATCC 19310^T	99.7	nd			*P. syringae*
FBF28	V	V	*P. rhodesiae* LMG 17764^T	91.7	nd			Putative new species
FBF30	V	V	*P. rhodesiae* LMG 17764^T	91.7	V	*P. marginalis* ATCC 10844^T / *P. grimontii* CIP 106645^T	94.9	Putative new species
FBF31	IV	IV	*P. lurida* P513/18^T	99.5	IV	*P. lurida* P513/18^T	99.7	*P. lurida*
FBF32	VI-A	VI-A	*P. syringae* ATCC 19310^T	97.5	nd			*P. syringae*
FBF33	VI-B	VI-B	*P. syringae* ATCC 19310^T	98	VI	*P. tremae* LMG 22121^T	97.1	*P. tremae*
FBF34	I	I	*P. orientalis* DSM 17489^T	97.8	nd			*P. orientalis*
FBF35	XI	XI	*P. japonica* JCM 21532^T	81.2	XI	*P. japonica* JCM 21532^T	91.9	Putative new species
FBF36	VI-B	VI-B	*P. syringae* ATCC 19310^T	98.2	VI	*P. tremae* LMG 22121^T	97.3	*P. tremae*
FBF38	V	V	*P. rhodesiae* LMG 17764^T	91.7	V	*P. marginalis* ATCC 10844^T / *P. grimontii* CIP 106645^T	94.9	Putative new species
FBF39	V	V	*P. rhodesiae* LMG 17764^T	91.7	nd			Putative new species
FBF40	VI-B	VI-B	*P. syringae* ATCC 19310^T	98	nd			*P. tremae*
FBF41	II	II	*P. libanensis* CIP105460^T	93.7	nd			Putative new species
FBF42	IV	IV	*P. lurida* P513/18^T	99.5	IV	*P. lurida* P513/18^T	99.7	*P. lurida*
FBF43	I	I	*P. orientalis* DSM 17489^T	97.8	nd			*P. orientalis*
FBF44	XI	XI	*P. japonica* JCM 21532^T	81.2	XI	*P. japonica* JCM 21532^T	91.9	Putative new species
FBF46	VI-D	VI-D	*P. syringae* ATCC 19310^T	99.7	VI	*P. syringae* ATCC 19310^T	99.4	*P. syringae*
FBF47	VI-D	VI-D	*P. syringae* ATCC 19310^T	99.8	nd			*P. syringae*

FBF48	I	I	*P. orientalis* DSM 17489^T	97.8	I	*P. orientalis* DSM 17489^T	97	*P. orientalis*
FBF49	VI-F	VI-B	*P. syringae* ATCC 19310^T	97.8	nd			*P. tremae*
FBF50	X	X	*P. monteilii* ATCC 700476^T	94	X	*P. monteilii* ATCC 700476^T	96.5	Putative new species
FBF51	I	I	*P. orientalis* DSM 17489^T	97.8	I	*P. orientalis* DSM 17489^T	97	*P. orientalis*
FBF52	VII	VII	*P. viridiflava* ATCC 13223^T	97.8	VII	*P. viridiflava* ATCC 13223^T	99.3	*P. viridiflava*
FBF53	V	V	*P. rhodesiae* LMG 17764^T	91.7	nd			Putative new species
FBF54	V	V	*P. rhodesiae* LMG 17764^T	91.7	V	*P. marginalis* ATCC 10844^T / *P. grimontii* CIP 106645^T	94.8	Putative new species
FBF55	V	V	*P. rhodesiae* LMG 17764^T	91.7	V	*P. marginalis* ATCC 10844^T / *P. grimontii* CIP 106645^T	94.9	Putative new species
FBF56	II	II	*P. synxantha* LMG 2335^T/ *P. veronii* LMG 17761^T	93.2	II	*P. synxantha* LMG 2335^T	96	Putative new species
FBF57	X	X	*P. monteilii* ATCC 700476^T	99	X	*P. monteilii* ATCC 700476^T	98.9	*P. monteilii*
FBF58	VIII	VIII	*P. syringae* ATCC 19310^T	85.4	VIII	*P. meliae* CCUG 51503^T / *P. tremae* LMG 22121^T	91.7	Putative new species
FBF59	V	V	*P. rhodesiae* LMG 17764^T	91.7	V	*P. marginalis* ATCC 10844^T / *P. grimontii* CIP 106645^T	94.9	Putative new species
FBF60	VI-B	VI-B	*P. syringae* ATCC 19310^T	98.1	nd			*P. tremae*
FBF61	VI-A	VI-A	*P. syringae* ATCC 19310^T	97.1	nd			*P. syringae*
FBF62	VI-A	VI-A	*P. syringae* ATCC 19310^T	97.8	nd			*P. syringae*
FBF63	VI-D	VI-D	*P. syringae* ATCC 19310^T	99.7	nd			*P. syringae*
FBF64	I	I	*P. orientalis* DSM 17489^T	97.8	I	*P. orientalis* DSM 17489^T	97	*P. orientalis*
FBF65	IX	IX	*P. moraviensis* DSM 16007^T	94.1	IX	*P. koreensis* LMG 21318^T	96.8	Putative new species
FBF66	I	I	*P. orientalis* DSM 17489^T	97.8	nd			*P. orientalis*
FBF67	IX	IX	*P. moraviensis* DSM 16007^T	93.6	nd			Putative new species
FBF68	VI-B	VI-B	*P. syringae* ATCC 19310^T	98	nd			*P. tremae*

FBF69	VI-A	VI-A	*P. syringae* ATCC 19310^T	97.8	nd			*P. syringae*
FBF71	VI-C	VI-C	*P. syringae* ATCC 19310^T	97.1	nd			*P. tremae*
FBF72	VI-C	VI-C	*P. syringae* ATCC 19310^T	97.4	nd			*P. tremae*
FBF73	V	V	*P. rhodesiae* LMG 17764^T	91.7	nd			Putative new species
FBF74	VI-C	VI-C	*P. syringae* ATCC 19310^T	97.5	nd			*P. tremae*
FBF75	V	V	*P. rhodesiae* LMG 17764^T	91.7	nd			Putative new species
FBF77	VI-B	VI-B	*P. syringae* ATCC 19310^T	98.2	nd			*P. tremae*
FBF78	VI-B	VI-B	*P. syringae* ATCC 19310^T	98.2	nd			*P. tremae*
FBF79	VI-B	VI-B	*P. syringae* ATCC 19310^T	97.5	nd			*P. tremae*
FBF80	I	I	*P. orientalis* DSM 17489^T	97.8	nd			*P. orientalis*
FBF81	I	I	*P. orientalis* DSM 17489^T	97.8	nd			*P. orientalis*
FBF82	VI-F	VI-B	*P. syringae* ATCC 19310^T	98.2	nd			*P. tremae*
FBF83	VI-B	VI-B	*P. syringae* ATCC 19310^T	97.7	nd			*P. tremae*
FBF84	I	I	*P. orientalis* DSM 17489^T	97.8	I	*P. orientalis* DSM 17489^T	97	*P. orientalis*
FBF85	IX	IX	*P. moraviensis* DSM 16007^T	94.1	nd			Putative new species
FBF86	I	I	*P. orientalis* DSM 17489^T	97.8	I	*P. orientalis* DSM 17489^T	97	*P. orientalis*
FBF87	IX	IX	*P. moraviensis* DSM 16007^T	94.1	nd			Putative new species
FBF88	IX	IX	*P. moraviensis* DSM 16007^T	94.1	nd			Putative new species
FBF89	IX	IX	*P. moraviensis* DSM 16007^T	94	nd			Putative new species
FBF90	IX	IX	*P. moraviensis* DSM 16007^T	94.1	nd			Putative new species
FBF91	VI-F	VI-B	*P. syringae* ATCC 19310^T	98	nd			*P. tremae*

FBF92	IX	IX	*P. moraviensis* DSM 16007^T	94.1	IX	*P. koreensis* LMG 21318^T	96.8	Putative new species
FBF93	V	V	*P. rhodesiae* LMG 17764^T	91.7	V	*P. marginalis* ATCC 10844^T / *P. grimontii* CIP 106645^T	94.8	Putative new species
FBF95	I	I	*P. orientalis* DSM 17489^T	97.8	nd			*P. orientalis*
FBF96	IX	IX	*P. moraviensis* DSM 16007^T	94.1	IX	*P. koreensis* LMG 21318^T	96.8	Putative new species
FBF97	VI-B	VI-B	*P. syringae* ATCC 19310^T	98.2	nd			*P. tremae*
FBF98	VI-C	VI-C	*P. syringae* ATCC 19310^T	97.5	nd			*P. tremae*
FBF99	IX	IX	*P. moraviensis* DSM 16007^T	94.1	IX	*P. moraviensis* DSM 16007^T / *P. koreensis* LMG 21318^T	96.2	Putative new species
FBF100	VII	VII	*P. viridiflava* ATCC 13223^T	91.8	VII	*P. viridiflava* ATCC 13223^T	95.6	Putative new species
FBF101	X	X	*P. monteilii* ATCC 700476^T	94	X	*P. monteilii* ATCC 700476^T	96.2	Putative new species
FBF102	VIII	VIII	*P. syringae* ATCC 19310^T	85	VIII	*P. meliae* CCUG 51503^T	91.7	Putative new species
FBF103	VIII	VIII	*P. syringae* ATCC 19310^T	85.4	VIII	*P. meliae* CCUG 51503^T / *P. tremae* LMG 22121^T	91.6	Putative new species
FBF104	VI-A	VI-A	*P. syringae* ATCC 19310^T	97.5	nd			*P. syringae*
FBF105	X	X	*P. monteilii* ATCC 700476^T	94	X	*P. monteilii* ATCC 700476^T	96.6	Putative new species
FBF106	VI-B	VI-B	*P. syringae* ATCC 19310^T	98.2	nd			*P. tremae*
FBF107	VI-C	VI-C	*P. syringae* ATCC 19310^T	97.5	nd			*P. tremae*
FBF108	VI-C	VI-C	*P. syringae* ATCC 19310^T	97.5	nd			*P. tremae*
FBF109	VI-E	VI-E	*P. syringae* ATCC 19310^T	98	nd			*P. syringae*
FBF110	III	III	*P. simiae* OLI^T	99.8	III	*P. simiae* OLI^T	99.96	*P. simiae*
FBF111	VI-D	VI-D	*P. syringae* ATCC 19310^T	99.7	nd			*P. syringae*
FBF112	III	III	*P. simiae* OLI^T	99.8	III	*P. simiae* OLI^T	99.96	*P. simiae*
FBF113	VI-B	VI-B	*P. syringae* ATCC 19310^T	97.5	nd			*P. tremae*

FBF114	X	X	*P. monteilii* ATCC 700476^T	94	X	*P. monteilii* ATCC 700476^T	95.8	Putative new species
FBF115	VI-B	VI-B	*P. syringae* ATCC 19310^T	96.8	nd			*P. tremae*
FBF116	VI-B	VI-B	*P. syringae* ATCC 19310^T	97.1	nd			*P. tremae*
FBF117	VII	VII	*P. viridiflava* ATCC 13223^T	97.7	nd			*P. viridiflava*
FBF118	VI-C	VI-C	*P. syringae* ATCC 19310^T	97.5	nd			*P. tremae*
FBF119	VI-A	VI-A	*P. syringae* ATCC 19310^T	97.5	nd			*P. syringae*
FBF120	III	III	*P. simiae* OLI^T	99.7	III	*P. simiae* OLI^T	99.96	*P. simiae*
FBF121	III	III	*P. simiae* OLI^T	99.8	nd			*P. simiae*
FBF122	VIII	VIII	*P. syringae* ATCC 19310^T	85.2	VIII	*P. meliae* CCUG 51503^T	91.6	Putative new species
FBF124	VI-B	VI-C	*P. syringae* ATCC 19310^T	97.8	VI	*P. tremae* LMG 22121^T	97.3	*P. tremae*
FBF125	VI-E	VI-E	*P. syringae* ATCC 19310^T	97.5	nd			*P. syringae*
FBF126	III	III	*P. simiae* OLI^T	99.8	nd			*P. simiae*
FBF128	III	III	*P. simiae* OLI^T	99.8	nd			*P. simiae*
FBF130	III	III	*P. simiae* OLI^T	99.8	nd			*P. simiae*
FBF134	VI-C	VI-C	*P. syringae* ATCC 19310^T	97.5	nd			*P. tremae*
FBF135	VI-E	VI-E	*P. syringae* ATCC 19310^T	97.2	VI	*P. syringae* ATCC 19310^T	98.6	*P. syringae*
FBF136	VI-B	VI-C	*P. syringae* ATCC 19310^T	97.8	VI	*P. tremae* LMG 22121^T	97.3	*P. tremae*
FBF138	VI-D	VI-D	*P. syringae* ATCC 19310^T	99.7	VI	*P. syringae* ATCC 19310^T	99.3	*P. syringae*
FBF139	VI-B	VI-B	*P. syringae* ATCC 19310^T	98.2	nd			*P. tremae*
FBF140	nd	II	*P. synxantha* LMG 2335^T	85.4	nd			Putative new species
FBF141	nd	II	*P. synxantha* LMG 2335^T/ *P. veronii* LMG 17761^T	92.5	nd			Putative new species
FBF142	nd	II	*P. synxantha* LMG 2335^T/ *P. veronii* LMG 17761^T	92.5	nd			Putative new species

FBF143	nd	II	*P. veronii* LMG 17761^T	92	nd	Putative new species
FBF144	nd	II	*P. synxantha* LMG 2335^T/ *P. veronii* LMG 17761^T	92.6	nd	Putative new species

nd, not determined
